# Real-time mapping of nanopore raw signals

**DOI:** 10.1093/bioinformatics/btab264

**Published:** 2021-07-12

**Authors:** Haowen Zhang, Haoran Li, Chirag Jain, Haoyu Cheng, Kin Fai Au, Heng Li, Srinivas Aluru

**Affiliations:** School of Computational Science and Engineering, Georgia Institute of Technology, Atlanta, GA, 30332, USA; Department of Biomedical Informatics, The Ohio State University, Columbus, OH, 43210, USA; Department of Computational and Data Sciences, Indian Institute of Science, Bangalore KA, 560012, India; Department of Data Science, Dana-Faber Cancer Institute, Boston, MA, 02215, USA; Department of Biomedical Informatics, Harvard Medical School, Boston, MA, 02215, USA; Department of Biomedical Informatics, The Ohio State University, Columbus, OH, 43210, USA; Department of Data Science, Dana-Faber Cancer Institute, Boston, MA, 02215, USA; Department of Biomedical Informatics, Harvard Medical School, Boston, MA, 02215, USA; School of Computational Science and Engineering, Georgia Institute of Technology, Atlanta, GA, 30332, USA; Institute for Data Engineering and Science, Georgia Institute of Technology, Atlanta, GA, 30332, USA

## Abstract

**Motivation:**

Oxford Nanopore Technologies sequencing devices support adaptive sequencing, in which undesired reads can be ejected from a pore in real time. This feature allows targeted sequencing aided by computational methods for mapping partial reads, rather than complex library preparation protocols. However, existing mapping methods either require a computationally expensive base-calling procedure before using aligners to map partial reads or work well only on small genomes.

**Results:**

In this work, we present a new streaming method that can map nanopore raw signals for real-time selective sequencing. Rather than converting read signals to bases, we propose to convert reference genomes to signals and fully operate in the signal space. Our method features a new way to index reference genomes using k-d trees, a novel seed selection strategy and a seed chaining algorithm tailored toward the current signal characteristics. We implemented the method as a tool Sigmap. Then we evaluated it on both simulated and real data and compared it to the state-of-the-art nanopore raw signal mapper Uncalled. Our results show that Sigmap yields comparable performance on mapping yeast simulated raw signals, and better mapping accuracy on mapping yeast real raw signals with a 4.4× speedup. Moreover, our method performed well on mapping raw signals to genomes of size >100 Mbp and correctly mapped 11.49% more real raw signals of green algae, which leads to a significantly higher *F*_1_-score (0.9354 versus 0.8660).

**Availability and implementation:**

Sigmap code is accessible at https://github.com/haowenz/sigmap.

**Supplementary information:**

Supplementary data are available at *Bioinformatics* online.

## 1 Introduction

Oxford Nanopore Technologies (ONT) sequencers produce millions of long reads with >10 kbp N50 in a single 48–72 h run. These long reads can span repetitive regions of a genome that are hard to resolve using short reads, thus enabling assemblies with high continuity (Miga et al., 2020). Direct RNA-sequencing through nanopores can sequence full-length RNA transcripts without amplification, which can greatly aid in *de novo* transcriptome analysis (Garalde et al., 2018). Without the need for additional library preparation, amplification-free nanopore sequencing also enables detection of nucleotide modifications (Simpson et al., 2017).

Nanopore sequencers work by measuring ionic current as a molecule passes through a pore. Since different molecules in the pore modulate the current in specific ways, individual nucleotides can be inferred by base calling of the raw current signal. For various ONT pore versions (e.g. R7, R9), the current signal is mainly affected by five or six nucleotides (i.e. *k*-mers where *k *=* *5 or 6) occupying the pore at a given time point. These current readings usually have a low signal-to-noise ratio, which makes it hard to identify the corresponding *k*-mers. To tackle this problem, many base callers have been developed to ‘translate’ the raw signals to nucleotide sequences (Rang et al., 2018). State-of-the-art base callers (e.g. ONT official base caller Guppy) can achieve around 90% accuracy. However, base calling is computationally expensive and can last days on a high-end central processing unit (CPU) or hours on a graphical processing unit (GPU) even for a relatively low throughput run with only ∼20 Gbp data.

The ONT MinION is a portable device that typically yields up to 30 Gbp sequencing data using a single flow cell at a low cost. Portability of the MinION sequencer allows sequencing to be performed in the field or the clinic, for example, surveillance for Ebola virus in West Africa (Quick et al., 2016) and fast detection of SARS-CoV-2 with high sensitivity (Wang et al., 2020). The MinION device is compatible with recently released Flongle flow cells with even lower prices while reducing the sequencing throughput to ∼2 Gbp for smaller analyses and tests. However, this throughput is usually too low for many applications that require high sequencing depth, which makes targeted sequencing necessary.

Targeted sequencing allows for enriched coverage of desired genomic regions, which reduces sequencing costs and labor to achieve high coverage at regions of interest. Typical targeted sequencing approaches do not work well with nanopore sequencing due to loss of nucleotide modifications, high input requirements, low throughput or long protocols (Gilpatrick et al., 2020). On the other hand, the targeted sequencing protocol designed specifically for nanopore sequencing (Gilpatrick *et al.*, 2020) addressed some of these issues, but still requires additional preparation time and is limited by the maximum size and number of targeted regions.

Alternatively, Loose et al. (2016) took advantage of the selective sequencing feature of the MinION sequencer and performed real-time targeted sequencing for amplicon enrichment. This is achieved by temporarily reversing the voltage across a nanopore, thereby rejecting an undesired molecule and making the pore available for other molecules. Thus, if there is a sufficiently fast computational method that can identify whether reads come from regions of interest, one can quickly eject undesired reads and leave the pores for reads of interest so that undesired genomic regions are not sampled and regions of interest are enriched. In their work, they use dynamic time warping (DTW) to align raw signals to reference genomes to decide whether reads are of interest. Since the time complexity of DTW is quadratic in terms of sequence length, it only works on small genomes that are kilobase pairs long. To address this issue, methods based on base calling followed by read mapping were proposed (Edwards et al., 2019; Payne et al., 2020). However, base callers are not optimized to work on small chunks of reads; thus, they may generate suboptimal read sequences, which makes mapping challenging (Kovaka et al., 2020). As base calling is a computationally intensive process, enough compute power (e.g. sufficiently powerful GPUs) to achieve real-time base calling may not always be available outside laboratories.

To avoid these drawbacks, Uncalled (Kovaka *et al.*, 2020) was developed to map raw signals in real time without base calling. It builds an FM-index (Ferragina and Manzini, 2005) for reference genomes, segments the raw signals into events (collapsed current readings for each *k*-mer) and converts the events into possible *k*-mers using the ONT pore model. High-probability *k*-mers are used to query the index and extended. Since raw signals are noisy, Uncalled keeps track of all possible positions of each *k*-mer as the mapping proceeds. After removing false-positive locations by a seed clustering method, the final mapping is reported if one of the locations is sufficiently better than the others. The authors demonstrated successful use of Uncalled on targeted sequencing of small genomes (<30 Mbp) and reported that it cannot work properly on mapping raw signals to large genomes that have high repeat content.

In this work, we present a new streaming method to map raw signals for real-time adaptive sequencing. In contrast to previous scalable methods, which convert signals to sequences and then leverage existing methods or data structures to map sequences, we convert reference genomes to signals and present a novel streaming method and tool Sigmap to map raw signals to the reference. We evaluated the performance of Sigmap and Uncalled on simulated and real data. Compared with Uncalled, while achieving comparable performance on mapping yeast simulated raw signals, Sigmap mapped slightly more yeast real raw signals accurately and provided 4.4× speedup. Moreover, Sigmap correctly mapped 11.49% more green algae raw signals with significantly higher *F*_1_-scores (0.9354 versus 0.8660). This indicates that our method can map raw signals to genomes of size >100 Mbp, an important advancement over previous base-calling-free methods.

## 2 Materials and methods

**Fig. 1.**
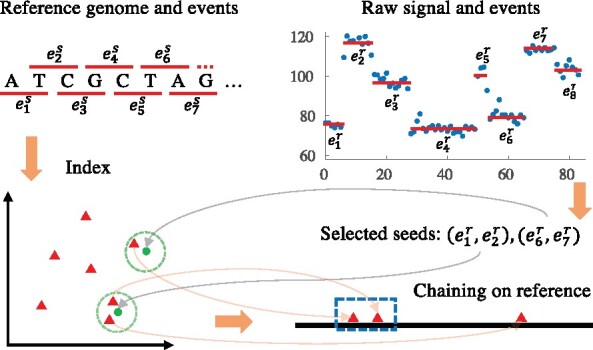
Overview of the proposed algorithm. The reference genome is first converted to a sequence of events $e_{1}^{s},e_{2}^{s},\ldots$ (red lines) using the expected current value of each *k*-mer in the pore model. For simplicity of illustration, we use 2-mers in this example. Now every pair of consecutive events $\left(e_{i}^{s},e_{i+1}^{s}\right)$ is a point in 2D space, thus a spatial index for these points (red triangles) can be created. For visualization purpose, we set dimension to 2, but higher dimensions may be used. In the mapping stage, raw signals (blue dots) are first segmented into events $e_{1}^{r},e_{2}^{r},\ldots$ (red lines). Then seeds are selected to query the index with range search and hits on the reference are chained to get the mapping (in the blue rectangle)

Seed-and-extend is a widely applied strategy to map erroneous long reads (Chaisson and Tesler, 2012; Li, 2018; Sedlazeck et al., 2018; Sović et al., 2016). Typically, exact or approximate word matches between reads and reference genomes are extracted and then co-linear matches (a sequence of matches that occur in ascending order in both reads and reference genomes) are identified to generate final alignments. Our algorithm also follows the seed-and-extend strategy (see Fig. 1 for an overview) but is specifically designed to handle noisy raw signal data. Prior to mapping, the reference genome is converted to events and an index of the reference is built once (Section 2.1). In the mapping step, raw current signals are first segmented into events and normalized (Section 2.2). Then seeds that are less likely to contain segmentation errors are selected from the processed raw signal and used to query the index (Section 2.3). After collecting the seed hits (anchors) on the reference, we designed and implemented a chaining algorithm tailored toward the current signal characteristics to find co-linear anchors as chains (Section 2.4). The chains are filtered by their scores to ignore suboptimal mappings. To do real-time selective sequencing, we presented a streaming version of the proposed algorithm (Section 2.5). The details of each step are as follows.

### 2.1 Indexing

Different pore models are provided by ONT for various pore versions since current readings are affected by different number of nucleotides occupying the pore at each sequencing time point. In this probabilistic model, current readings for each *k*-mer are assumed to follow a Gaussian distribution with known parameters. Thus using the pore model, one can estimate the probability of a given event being any of the *k*-mers, or convert a nucleotide sequence to an event sequence by simply substituting *k*-mers with their expected current readings.

Uncalled uses the prior strategy to generate high-probability *k*-mers from read events, while our method leverages the latter to convert the reference to events. Note that in the first case, a full iteration on all the distributions is usually required to identify high-probability *k*-mers that an event may correspond to, which can be slow when many events in the read are processed simultaneously. But converting a *k*-mer to its expected current reading is a direct translation once a hash table is built for the pore model using *k*-mers as keys and expected current as values. Since the conversion is only done once for reference genomes, we can save the overhead of applying pore models to read events to find high-probability *k*-mers during the mapping stage.

Formally, let $s=s_{1}s_{2}\ldots s_{n}$ be a nucleotide sequence of length *n* over alphabet Σ and its corresponding sequence of *k*-mers be $K(s)=k_{1}k_{2}\ldots k_{n-k+1}$, where $k_{i}=s_{i}s_{i+1}\ldots s_{i+k-1}$. The pore model is defined as $f:\Sigma^{k}\overset{}{\to }R$, which gives the expected current corresponding to a *k*-mer. We create the corresponding event sequence as $E(s)=e_{1}^{s}e_{2}^{s}\ldots e_{n-k+1}^{s}$, where $e_{i}^{s}=f(k_{i})$. This is translated to a set of points $P(s)=\{p_{i}^{s}=(e_{i}^{s},e_{i+1}^{s},\ldots ,e_{i+d-1}^{s}),1\leq i\leq n-k-d+2\}$ in *d*-dimensional space. Similarly, for each raw signal sequence *r*, we generate its events $E(r)=e_{1}^{r}e_{2}^{r}\ldots e_{m}^{r}$ (described in Section 2.2). The reads are also translated to points $P(r)=\{p_{i}^{r}=(e_{i}^{r},e_{i+1}^{r},\ldots ,e_{i+d-1}^{r}),1\leq i\leq m-d+1\}$ in *d*-dimensional space, some of which are used as seeds in the mapping step. Therefore, we need a data structure to organize points of the reference sequence in *d*-dimensional space so that given a query point *p^r^* of the read, we can efficiently retrieve points $p_{i_{1}}^{s},p_{i_{2}}^{s},\ldots$ of the sequence near *p^r^*, i.e. ${\left\|p^{r}-p_{i_{j}}^{s}\right\|}_{2}\leq \epsilon$ where *ϵ* is the threshold for this range search.

The k-d tree (Bentley, 1975) is a data structure designed for partitioning space and organizing points with a binary tree. The leaf nodes of the tree are points while every non-leaf node implicitly divides a subspace into two parts by a hyperplane within that subspace. The points on either side of this hyperplane are associated with the left/right subtrees, respectively. In a balanced k-d tree, the time complexity of range search is $O(dn^{1-\frac{1}{d}})$ in worst case for a fixed range size (Lee and Wong, 1977). But in practice, this typically takes $O(\text{log}\;n+2^{d})$ time, where logarithmic time is spent in finding the nodes ‘near’ the query point and $O(2^{d})$ time is spent to explore their neighborhoods. Therefore, we use the k-d tree to organize points generated from the reference to handle large number of queries efficiently during mapping process. Note that construction of the index requires O(n log n) time when using an *O*(*n*) median of medians algorithm (Cormen et al., 2009), and the index only needs to be built once prior to mapping. In the implementation, we used the highly optimized k-d tree package nanoflann (https://github.com/jlblancoc/nanoflann), which supports k-d tree construction and queries.

### 2.2 Signal pre-processing

There are two signal pre-processing steps: signal segmentation and normalization. For R9.4 pore, the DNA molecule transits through the pore with an average speed of 450 bp/s and the electric current is sampled at 4 kHz, which means on average each *k*-mer has around eight current samples. The purpose of signal segmentation is to collapse the current readings of the same *k*-mer into an event. However, speed of the molecule passing through the pore varies significantly. As a result, some *k*-mers may stay longer in the pore and generate more current readings (*stay errors*) while some *k*-mers may have no recorded current as the time they reside in the pore is too short (*skip errors*), which makes it hard to segment signals accurately. Moreover, to process signals in real time, we need a fast segmentation method.

Scrappie (https://github.com/nanoporetech/scrappie) is a base caller from ONT, which has a segmentation step prior to fine-grained base calling. It uses *t*-test over rolling window on the raw signal to detect where the current changes significantly, thereby segmenting the signal. Similar to this method, we also use the Welch’s *t*-test to segment the signal. We choose a fixed window size *w* and for raw current samples in every two adjacent windows we compute the t-statistics $t=({\bar{x}}_{1}-{\bar{x}}_{2})/\sqrt{(y_{1}^{2}+y_{2}^{2})/w}$ where ${\bar{x}}_{i}$ is the current sample mean and *y_i_* is the current sample standard deviation in the window. Then all the local maxima and minima are identified among the computed t-statistics along the sequence. When a local extremum passes a significance threshold, its position is selected to segment the signal. Due to the various molecule transiting speeds, t-statistics should be computed using multiple window sizes. Local extrema are chosen as segmentation positions using the smallest possible window size if the local extrema reach the significance threshold of that window size. After the signal is segmented, the detected events are normalized to account for the shift or drift during sequencing.

### 2.3 Seeding

After reference genomes and raw signals are converted into events, the mapping problem is as follows: given read events *E*(*r*) and reference events *E*(*s*), find consecutive events $E_{i,j}(s)=e_{i}^{s}e_{i+1}^{s}\ldots e_{j}^{s}$ in *E*(*s*) such that *E*(*r*) can be aligned to $E_{i,j}(s)$ with high confidence. Note that the mapping can be found by using subsequence dynamic time warping (sDTW) (Han et al., 2020). But the time to compute DTW distance is quadratic in the length of event sequences, which is too slow to compute for long reads in real time. Since the reads are long, though they are erroneous, there are still many subsequences shared in a high confidence mapping region of the read and the reference. Taking advantage of this fact, long-read aligners such as minimap2 (Li, 2018) can efficiently map reads using the seed-and-extend strategy and so does our method.

As the reference points are indexed for fast queries, we can use read points $P(r)=\{p_{i}^{r}=(e_{i}^{r},e_{i+1}^{r},\ldots ,e_{i+d-1}^{r}),1\leq i\leq m-d+1\}$ as the seeds. Note that the number of seeds (or points) needed to query the index is roughly the length of the event sequence. For real-time mapping, the reads have to be mapped within their first few hundreds of base pairs (events). Thankfully, searching for all the seeds can be completed in reasonable time. However, more seeds also lead to more hits on the reference, thereby potentially increasing the time spent in chaining the hits. For organisms like yeast, the number of hits is limited by the small genome size and fewer repetitive regions. But for larger genomes with more repetitive structures, the number of hits can increase significantly, which makes the chaining step time-consuming.

To address this problem, one can select seeds with a fixed step size *l* and only use a subset of all the read points *P*(*r*) as seeds, $P_{l}(r)=\{p_{i}^{r}=(e_{i}^{r},e_{i+1}^{r},\ldots ,e_{i+d-1}^{r}),1\leq i\leq m-d+1,i\;\mathrm{mod}\;l=0\}$. However, raw signals are noisy, which also makes the events erroneous. Simply picking seeds with a fixed step size could miss some ‘error free’ seeds (query points that have true hits in the index within a certain range) and reduce mapping accuracy. This problem is even more serious when mapping reads in a streaming manner, where the read is supposed to be mapped with only its first few hundreds of base pairs sequenced.

As an alternative, if the quality of the seed can be measured by a score, then error-free seeds can be preferred during seed selection procedure. Formally, we define a scoring function $g:R^{d}\overset{}{\to }R$, which computes the score for a given point in *d*-dimensional space. Note that during sequencing, stay errors happen more frequently than skip errors. Affected by the noise during sequencing, stay errors result in many current samples for the same *k*-mer with large variance, which leads to over segmentation of the raw signal. If a seed contains stay errors, range search can fail to find true hits of the seed.

We present a method to avoid seeds that are likely to contain stay errors. For a seed (query point) $p_{i}^{r}=(e_{i}^{r},e_{i+1}^{r},\ldots ,e_{i+d-1}^{r})$, we define the seed scoring function as $g(p_{i}^{r})=\sum_{j=i+1}^{i+d-1}\left|e_{j}^{r}-e_{j-1}^{r}\right|$, which is the sum of the differences between every pair of consecutive events in the seed. Then with step size *l*, top ⌈(m−d+1)/l⌉ seeds are selected based on their scores. Note that seeds with more abrupt changes in their events are considered better since the segmentation is more reliable in that case.

### 2.4 Chaining

The time for computing an optimal alignment between two sequences is quadratic in the length of the sequences. To avoid this computational bottleneck for aligning long sequences, chaining approaches (Li, 2018) have been proposed and used to efficiently find mapping positions of long reads in large reference genomes.

Inspired by the chaining method of minimap2, we present a dynamic programming algorithm to identify a set of co-linear anchoring point matches. Formally, each seed hit (anchor) is a triple (*u*, *v*, *h*), which represents a read point $p_{u}^{r}$ matching a reference point $p_{v}^{s}$ with distance *h*, i.e. ${\left\|p_{u}^{r}-p_{v}^{s}\right\|}_{2}=h$. Given a list of anchors sorted by their positions on the reference, the best chaining score up to the *i*th anchor can be computed using the recurrence $D_{i}=\max\left\{\max_{1\leq j<i}\left\{D_{j}+\alpha_{ji}-\beta_{ji}\right\},(1-h_{i}/\epsilon )d\right\}$, where $\alpha_{ji}=(1-h_{i}/\epsilon )*\min\left\{u_{i}-u_{j},v_{i}-v_{j},d\right\}$ is the bonus for the seed hit and *β_ji_* is the gap penalty. Let $a_{ji}=\left|\left(u_{i}-u_{j})-(v_{i}-v_{j}\right)\right|$ denote the gap length and $b_{ji}=\left|\left(u_{i}-u_{j})/(v_{i}-v_{j}\right)\right|$ denote the gap scale. The gap penalty *β_ji_* is set to ∞ when *v_i_* < *v_j_* (*i*th anchor is not co-linear with the *j*th anchor), or gap length *a_ji_* or gap scale *b_ji_* is too large. Due to stay and skip errors, the gap length and scale are usually unpredictable. Hence, we do not penalize the gap as long as its length and scale are below certain thresholds. Instead, when computing the bonus *α_ji_* for seed hits, we scale it down by the factor $\left(1-h_{i}/\epsilon \right)$.

Note that the time of the chaining algorithm is quadratic in the number of anchors, which is slow. In practice, we use similar heuristics as in minimap2 chaining to reduce the number of anchors to examine. When computing *D_i_*, we start the iteration from j=i−1 and stop when no better chaining score is found after *c* iterations. For *n_a_* anchors, this heuristic reduces the average time to $O(cn_{a})$. The default *c* is set to the same value used in minimap2 since it led to reasonable speed and accuracy on mapping reads to various genomes empirically. There are theoretically faster chaining algorithms (Abouelhoda and Ohlebusch, 2005) but they are usually not adapted to generic gap functions, or have large hidden constants in their time complexity.

### 2.5 Streaming mapping

In nanopore real-time sequencing, the signal is returned in chunks, and each chunk by default is one second’s worth of signal and contains 4000 current samples or roughly 450 bp. We developed a streaming method to map raw signals by chunks. The signal preprocessing and seeding are performed on each chunk individually. As for chaining, the anchors in the good chains (chaining scores are at least half of the best score) generated using previous chunks are kept and used in the chaining together with the anchors in the current chunk. Each time after a chunk is processed, we compute the ratio between the best chaining score and the second best chaining score. If the ratio exceeds a certain threshold, we stop mapping more chunks and report the best chain as the mapping. By default, we set this ratio to 1.4. If this ratio cannot exceed this threshold after mapping the first 30 chunks of the read, the mapping process of this read will be stopped and the read will be reported as unmapped. These parameters can be adjusted by users to increase mapping speed or lower false-positive rate based on the applications if necessary.

## 3 Experimental results

We demonstrate empirically the advantages of our method on both simulated and real datasets on two different genomes. The implementation of our proposed method is termed *Sigmap*, which is available at https://github.com/haowenz/sigmap. We compare Sigmap with Uncalled (v2.1).

### 3.1 Experimental setup

#### 3.1.1 Benchmarking datasets

We used one simulated and two real datasets to test the methods. The number of reads, N50 values, genome sizes and average coverage for these datasets are shown in Table 1. Simulated raw signals of *Saccharomyces cerevisiae* (yeast) were generated using DeepSimulator (Li et al., 2020) with its context-dependent model (-M 0) and sequencing coverage set to 20× (-K 20). For real datasets, 100 000 raw reads were randomly selected from nanopore sequencing of *S.cerevisiae* using ONT R9.4 chemistry (available at NCBI under the study PRJNA510813). The first run of *Chlamydomonas reinhardtii* (green algae) nanopore sequencing using ONT R9.4 chemistry was also used (under study PRJEB31789 on EMBL-EBI) in the evaluation. Note that in real-time targeted sequencing applications, the regions of interest are usually from ∼10 to ∼100 Mbp and the coverage of target regions is around 20× (Kovaka *et al.*, 2020; Miller et al., 2020). Thus in the evaluation, the yeast and green algae sequencing data were used as their genome sizes are appropriate and their whole-genome sequencing data are subsampled to the proper coverage for real-time targeted sequencing applications. Besides, since Uncalled only supports R9.4 chemistry so far, we used R9.4 data in our evaluation. But with some parameter tuning for both methods, they might also be able to work on R10 data with the R10 pore model (https://github.com/jts/nanopolish/tree/r10/etc/r10-models) trained using Nanopolish (Simpson *et al.*, 2017).

**Table 1.** List of benchmarking datasets

Dataset	Type	Number of reads	N50 (bp)	Reference genome	Genome size (Mbp)	Avg. coverage
D1	Simulated	30 385	11 984	*S.cerevisiae* S288c	12.2	20×
D2	Real	100 000	8348	*S.cerevisiae* S288c	12.2	58×
D3	Real	63 215	32 025	*C.reinhardtii* v5.5	111.1	12×

#### 3.1.2 Hardware and software

For all experiments, we used a compute node with dual Intel Xeon Gold 6226 CPU (2.70 GHz) processors equipped with a total of 24 cores and 128 GB main memory. We run Sigmap and Uncalled with all the available cores.

The k-d tree index constructed by Sigmap has two important parameters: dimension *d* and the maximum number of points associated with a leaf node, *n_p_*. The empirical performance of k-d trees is usually good in low-dimensional spaces (e.g. 2D or 3D) but degrades in high-dimensional spaces as more tree branches need to be visited for each query. For this application, a low *d* such as 2 or 3 cannot be chosen, as querying points in low-dimensional spaces usually results in too many hits, which can slow down mapping. Thus, we set *d* to 6 by default. Since the ONT R9.4 pore model lists the expected current reading for each 6-mer, a point in the 6-dimensional space is analogous to an 11-mer, which is also a reasonable *k*-mer size for read mapping on genomes from tens of Mbp to several hundred Mbp. As for the other parameter, *n_p_* controls the maximum number of points associated with a leaf node (points are stored in leaf nodes of k-d trees). A larger *n_p_* can make the tree smaller but may cause more explorations of points during the search process and increase the query time. On the other hand, a smaller *n_p_* may reduce the number of points to inspect for a query but increase the tree size. By default, we set *n_p_* to 20 and studied how it can affect memory usage and mapping time on D2. Moreover, to study the effect of seeding step size on mapping time, we evaluated Sigmap with various seeding step sizes *l* from 2 to 6 on D3 while other parameters are set to the default. We set the maximum amount of chunks to use for mapping a raw signal as 30 and the search radius *ϵ* to 0.08 by default since they led to proper mapping accuracy and time. These parameters can be adjusted by users according to their data and applications in practice.

To test Uncalled, we used default parameters for indexing reference genomes and mapping raw signals. Kovaka *et al.* (2020) showed that masking repeats in genomes improved the mapping speed and accuracy of Uncalled. In the evaluation, we used recommended parameters and procedures stated in the Uncalled’s user documentation for *C.reinhardtii* genome repeat masking.

#### 3.1.3 Evaluation criteria

We followed a similar evaluation criteria previously used by Kovaka *et al.* (2020). Raw reads that are mapped to their true mapping locations are true positives (TP). Reads that are mapped by their raw signals but not to the correct locations are false positives (FP). Reads that have true mapping locations but are not mapped by their raw signals are false negatives (FN). Precision equals TP/(TP+FP), recall equals TP/(TP+FN) and *F*_1_-score is calculated by 2*precision*recall/(precision+recall). The percent of correctly mapped reads is the portion of reads that are mapped to their true mapping locations.

For simulated dataset D1, we evaluated the mapping accuracy against the ground truth output by the simulator. For real datasets, we mapped the base-called read sequences with the well-established long-read aligner minimap2 (Li, 2018) and used the read alignments as ground truth to validate Sigmap and Uncalled. We excluded reads that are not mapped by minimap2 in the evaluation.

Moreover, we measured the mean mapping time of each read and the number of chunks used to map a read. In practical applications, mapping results are needed in real time to decide whether to eject a pore. Therefore, instead of cumulative mapping time, time spent on individual reads is an important metric to show whether most of the reads can be mapped fast enough for real-time decisions. To accurately measure the mapping time for individual reads, the mapping start time and end time of each read were recorded and the wall time for mapping each read was computed as the difference between these two values and then reported. This way of timing the mapping process for individual reads avoids the effect of loading index or the scalability of multithread implementation on measuring mapping time, which is a fair way to compare the two methods.

### 3.2 Comparison with Uncalled

We evaluated the performance of Sigmap and Uncalled on datasets D1–D3. The results on yeast genome are shown in Table 2. On the simulated dataset D1, Sigmap achieved higher percentage of correctly mapped reads, precision and *F*_1_-score while Uncalled has higher recall and faster speed. Since simulated data might not be as noisy as real data, the events were likely to be detected and converted to corresponding *k*-mers more reliably, which reduced the number of high-probability *k*-mers to explore in Uncalled and made it faster. On yeast real dataset D2, 93 544 of the 100 000 reads were mapped by minimap2 and used in the evaluation. Sigmap achieved higher percent of correctly mapped reads, precision, recall and *F*_1_-score. Notably, its speed of mapping a raw signal on average was 4.4 times faster than Uncalled.

**Table 2.** Performance comparison between Sigmap and Uncalled on yeast genome

Dataset	Method	Correctly mapped reads (%)	TP	FP	FN	Precision (%)	Recall (%)	F1-score	Mean time per read (ms)
D1	Sigmap	97.66	29675	7	661	99.98	97.82	0.9889	59
	Uncalled	97.47	29615	722	47	97.62	99.84	0.9872	18.3
D2	Sigmap	87.54	81892	964	10683	98.84	88.46	0.9336	68.3
	Uncalled	87.37	81725	1054	10765	98.73	88.36	0.9326	303.1

The best numbers are highlighted in bold.

Next, we tested Sigmap and Uncalled on the green algae real dataset D3, where minimap2 mapped 60 313 out of 63 215 reads. Table 3 shows the evaluation results. We denote Sigmap run with seeding step size 3 by Sigmap (l3), etc. Since the green algae genome is much larger than the yeast genome and has more repetitive regions, genome repeat masking was performed as suggested when using Uncalled to map raw signals. After repeat masking, both mapping accuracy and mean time to map a read improved. But Sigmap significantly outperformed Uncalled with or without repeat masking on the percentage of correctly mapped reads, recall and *F*_1_-score, while achieving comparable precision. Moreover, compared with Uncalled with and without masking, respectively, Sigmap using default parameters was 1.3 and 1.2 times faster on mapping reads, and Sigmap using seeding step size 6 was 2.6 and 2.3 times faster. Though the mapping accuracy of Sigmap degraded when increasing the seeding step size, it was overall better compared to Uncalled. The reason for this observation is that using larger seeding step size reduces the number of picked seeds that go into chaining, which would reduce chaining time and thereby reducing mapping time. But picking fewer seeds also reduced the mapping accuracy since the true mapping location would have fewer supported seeds making it harder to distinguish from other false mapping locations.

**Table 3.** Performance comparison between Sigmap and Uncalled on green algae genome

Dataset	Method	Correctly mapped reads (%)	TP	FP	FN	Precision (%)	Recall (%)	F1-score	Mean time per read (ms)
D3	Sigmap	87.86	52989	1694	5628	96.90	90.40	0.9354	509.1
	Sigmap (l3)	86.21	51998	1973	6338	96.34	89.14	0.9260	373
	Sigmap (l4)	83.51	50370	2542	7397	95.20	87.20	0.9102	314.8
	Sigmap (l5)	80.69	48669	3107	8532	94.00	85.08	0.8932	279.6
	Sigmap (l6)	77.20	46564	3781	9962	92.49	82.38	0.8714	261.2
	Uncalled	72.18	43534	883	15896	98.01	73.25	0.8384	677
	Uncalled (mask)	76.37	46060	881	13372	98.12	77.50	0.8660	596.5

The best numbers are highlighted in bold.

**Fig. 2.**
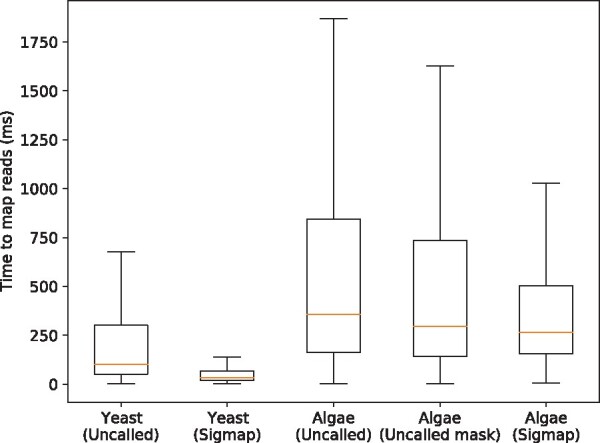
Boxplots showing the mapping time distributions of Uncalled and Sigmap on mapping real reads in D2 and D3. Center lines denote the median, box limits are the quartiles and the whiskers extended from the boxes represent 5% and 95% confidence intervals

**Fig. 3.**
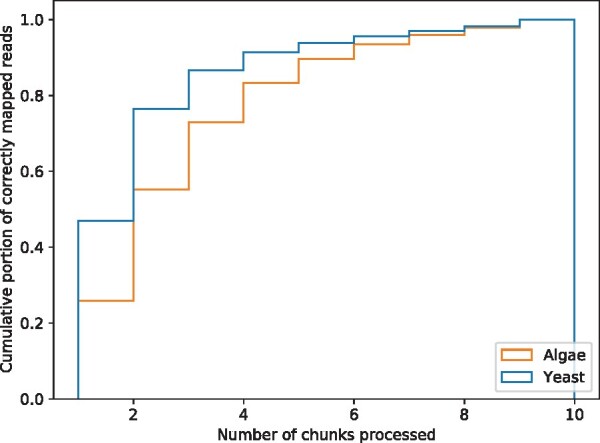
Number of chunks processed by Sigmap to correctly map reals read in D2 and D3. Most of the reads were mapped using ≤10 chunks

The mapping time distributions of Uncalled and Sigmap on D2 and D3 are shown in Figure 2. We observed that overall Sigmap achieved much shorter mapping time on mapping yeast real raw reads compared with Uncalled. We noticed the speedup of mapping reads on green algae genome is not as significant as the speedup of mapping yeast reads. One reason is that the size of green algae genome is as around nine times larger as the size of yeast genome. Given the fact that in practice the time of k-d tree queries is usually logarithmic in the number of points (explained in Section 2.1), which is roughly the size of the genome, the query time is supposed to increase accordingly. In addition, the green algae genome has more repetitive regions than the yeast genome and thus the number of signal chunks needed to map algae reads confidently on average is expected to be greater than that to map yeast reads. In the evaluation, we studied the number of chunks needed for Sigmap to map yeast and green algae reads correctly and present the results in Figure 3. We observe that using the same number of chunks, a smaller fraction of green algae reads were correctly mapped compared with yeast reads. This also indicates overall more chunks were needed to map green algae reads confidently, which increased the mapping time.

Besides mapping speed, we investigated the index size of Sigmap and Uncalled, which contributes to most of the memory usage in real-time signal mapping. Note that Uncalled mainly relies on an FM-index of the reference sequence, which is a compressed full-text index, hence expected to be space-efficient. The index size of the yeast genome and the green algae genome built by Uncalled is 21 and 186 MB, respectively. Using default parameters, Sigmap built a 417 MB index for the yeast genome and a 3.2 GB index for the green algae genome, which are larger than the indices built by Uncalled but can still be accommodated on typically used computing systems.

**Fig. 4.**
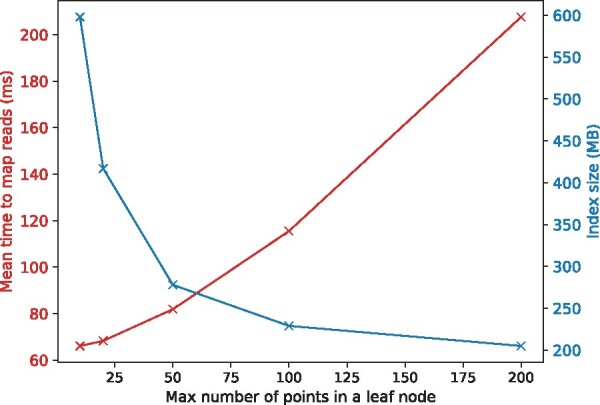
Index size and mean read mapping time with respect to the maximum number of points allowed in a leaf node of the k-d tree

As discussed in Section 3.1.2, increasing the maximum number of points associated with a leaf node, *n_p_*, can trade off mapping speed for smaller index. We studied how mean time to map reads and index size vary with different $n_{p}=10,20,50,100,200$ on D2 and showed the results in Figure 4. We observed that the mean mapping time increased and the index size decreased as *n_p_* increased and when *n_p_* = 200, the index size can be reduced by a half while the average time to map a read increased by about two times. Similarly, the index size of green algae genome can be reduced to 1.8 GB when setting *n_p_* = 200.

Note that the space complexity of the k-d tree is linear in the number of points and the reason that Sigmap index size is large can be partly attributed to the implementation. Therefore, another possible way to reduce the index size without sacrificing mapping speed is to implement a memory efficient k-d tree customized for this application rather than using a generic k-d tree library, which is a useful direction for future work.

## 4 Conclusions

Mapping nanopore raw signals in real time is challenging under limited computing resources. Most mapping methods require base calling, which is computationally expensive. Uncalled is an efficient method that does not require base calling, but hits performance limitations on large genomes with higher repeat content. In this work, we introduced a new nanopore raw signal mapping method and implemented it as a tool Sigmap. On small genomes like yeast, while Sigmap has comparable performance with Uncalled on mapping simulated data, Sigmap is 4.4× faster than Uncalled on mapping yeast real raw signals and has the potential to support real-time signal mapping for high-yield run ONT sequencing devices with more pores (e.g. GridION), which previous mapping methods without base calling might not be able to achieve. Sigmap also has good performance on genomes of size >100 Mbp such as green algae, where Uncalled could not identify many correct mappings. The method avoids any conversion of signals to sequences and fully works in signal space, which holds promise for completely base-calling-free nanopore sequencing data analysis.

We envision two directions for future research. First, we intend to accelerate Sigmap by utilizing CPU SIMD instruction sets or GPUs so that it can scale to support real-time sequencing on GridION or PromethION, which has even more pores. Second, we plan to study whether Sigmap can be adapted to map RNA nanopore raw signals. This may require the development of new seeding and chaining methods that are suitable to the characteristics of direct RNA-sequencing.

## Funding

This work is supported in part by the US National Science Foundation under CCF-1816027, the National Human Genome Research Institute [grant number R01HG010040] and the National Institutes of Health [grant number R01HG008759]. It is also supported in part by an institutional fund of the Department of Biomedical Informatics, the Ohio State University and an institutional fund of the Department of Internal Medicine, University of Iowa.

*Conflict of Interest*: H.L. is a consultant of Integrated DNA Technologies and on the Scientific Advisory Boards of Sentieon, BGI and OrigiMed.
